# Supplementary material for: Early Detection of Both Pyrenophora teres f. teres and f. maculata in Asymptomatic Barley Leaves Using Digital Droplet PCR (ddPCR)
Source: Int J Mol Sci. 2024 Nov 7;25(22):11980. doi: 10.3390/ijms252211980 (PMC11593351; doi:10.3390/ijms252211980)
Supplement: Supplementary file 1 [file ijms-25-11980-s001.zip › ijms-3261379-supplementary.pdf]

SUPPLEMENTARY DATA

Early detection of both *Pyrenophora teres* f. *teres* and f. *maculata* in asymptomatic barley leaves by droplet digital PCR (dPCR)

Yassine Bouhouch[1,2], Dina Aggad[2], Nicolas Richet[1#], Sajid Rahman[4], Muamar Al-Jaboobi[4], Zakaria Kehel[4], Qassim Esmaeel[1], Essaïd Aït Barka[1], Majida Hafidi[3], Cédric Jacquard[1], Lisa Sanchez[1]

Neupane A, Tamang P, Brueggeman RS, Friesen TL. 2015. Evaluation of a barley core collection for spot form net blotch reaction reveals distinct genotype-specific pathogen virulence and host susceptibility. Phytopathology. 105(4):509-17. doi: 10.1094/PHYTO-04-14-0107-R. PMID: 25870926.

Tekauz, A. 1985. A numerical scale to classify reactions of barley to *Pyrenophora teres*. Can. J. Plant Pathol. 7: 181-183.

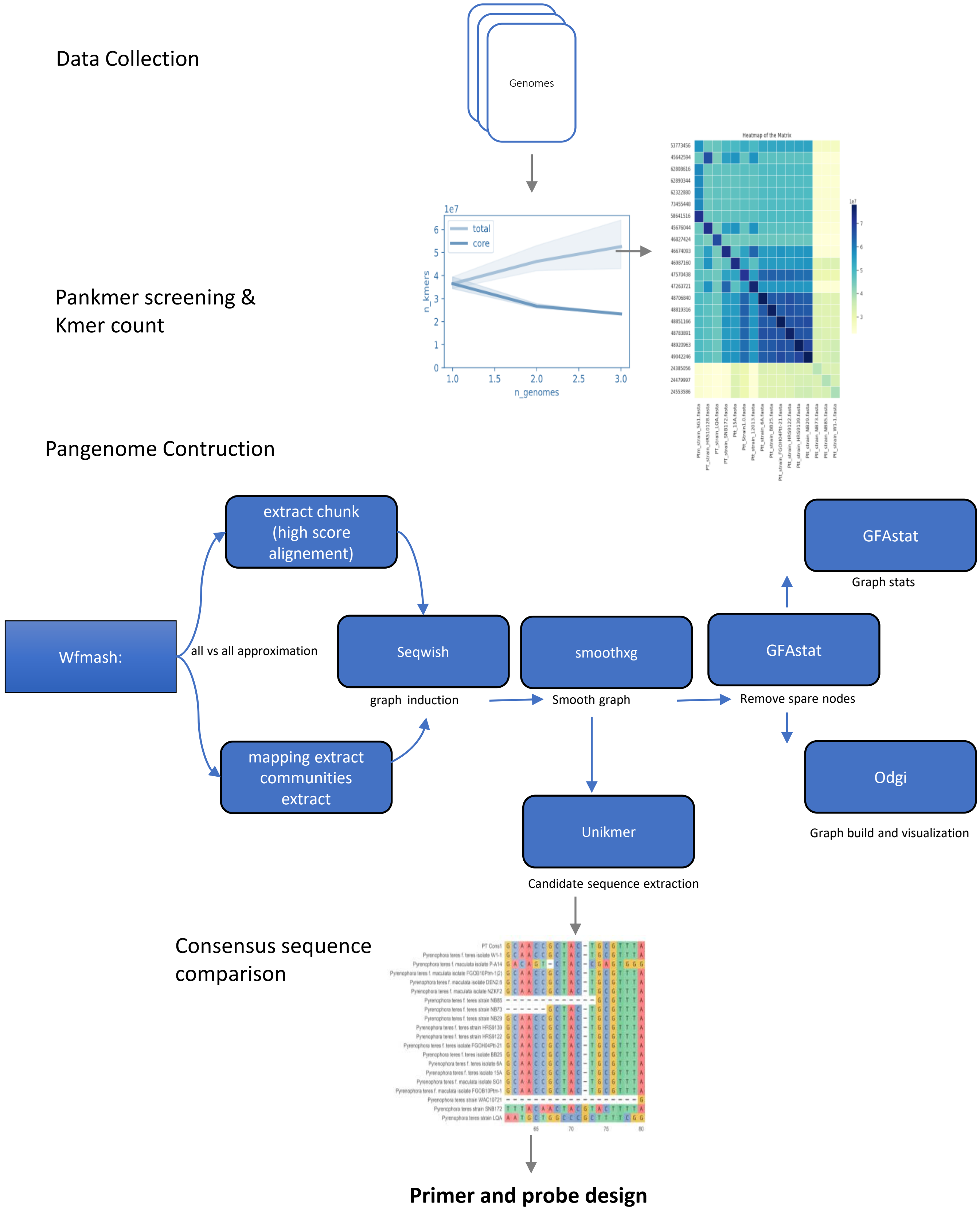

Supplementary figure S1: Worflow used in this study to define consensus primers and probe

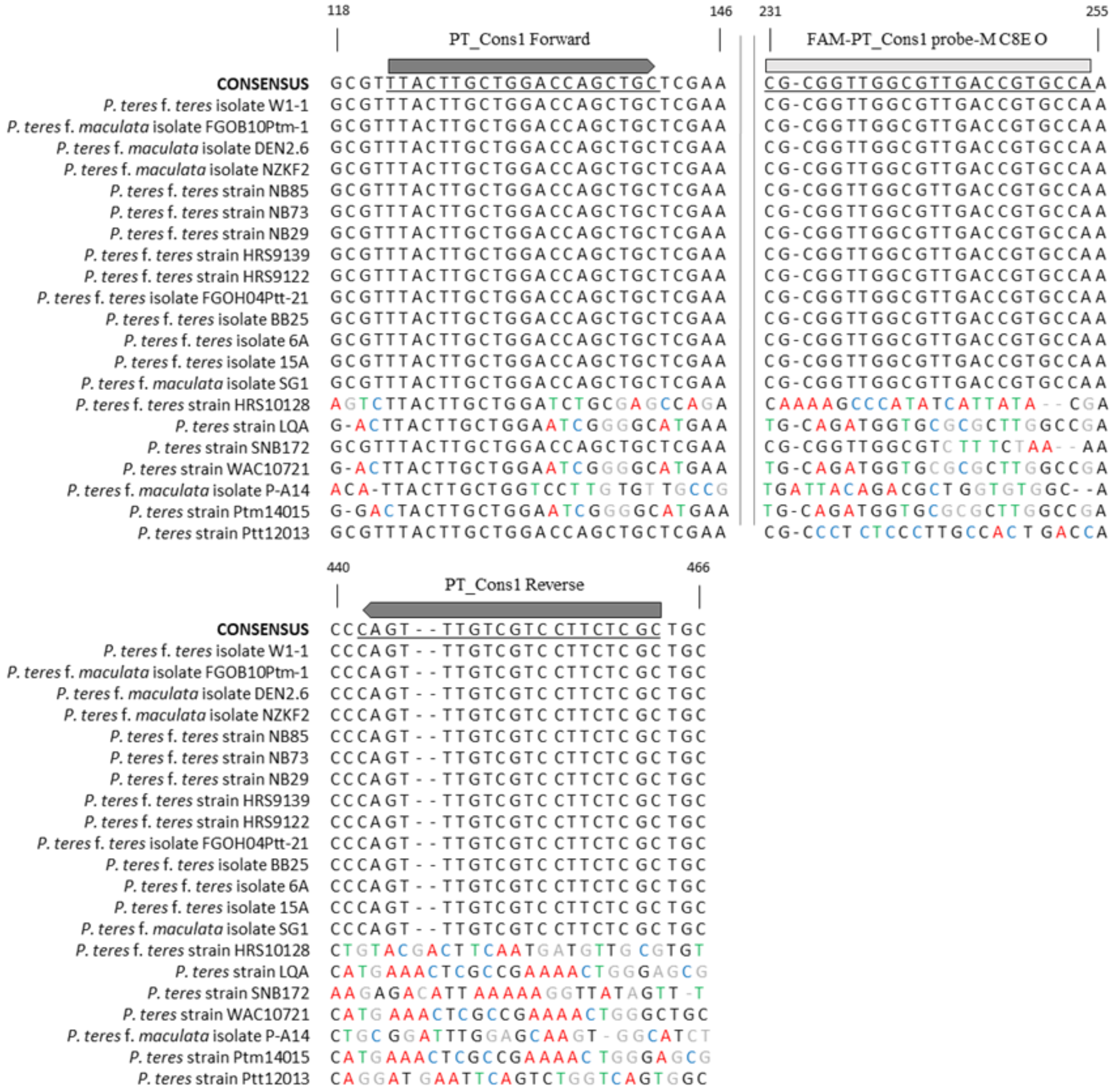

Supplementary figure S2: Multiple sequence alignment of the PT_Cons1 region from available *Pyrenophora teres* genomes. The consensus sequence is displayed at the top, with individual isolates' sequences aligned below. Nucleotide positions where the isolates differ from the consensus are highlighted in different colors, with the color corresponding to the specific nucleotide substitution. White boxes represent nucleotide identity to the consensus sequence, while dashes indicate sequence gaps.

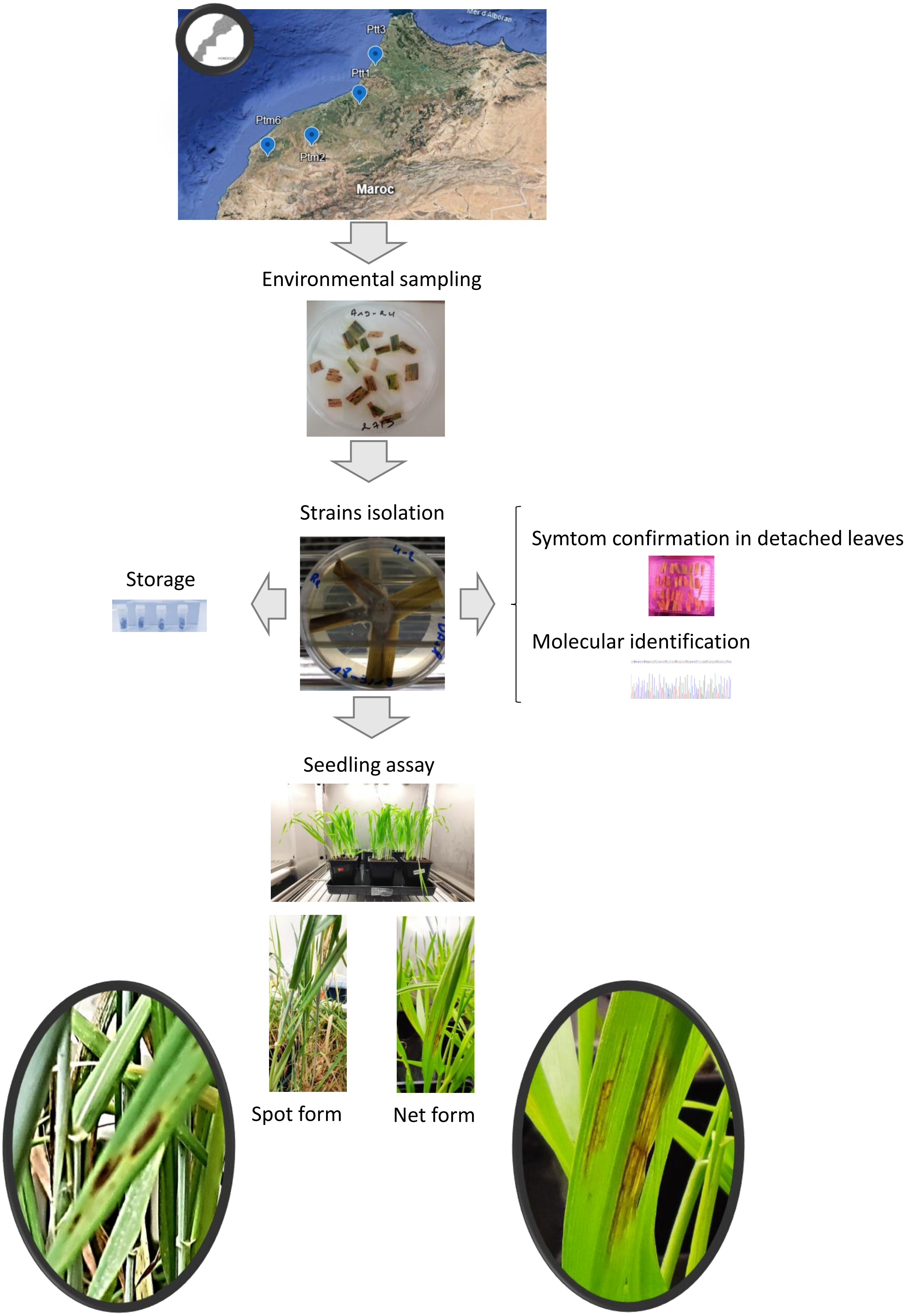

Supplementary figure S3: Isolation of moroccan fungal strains

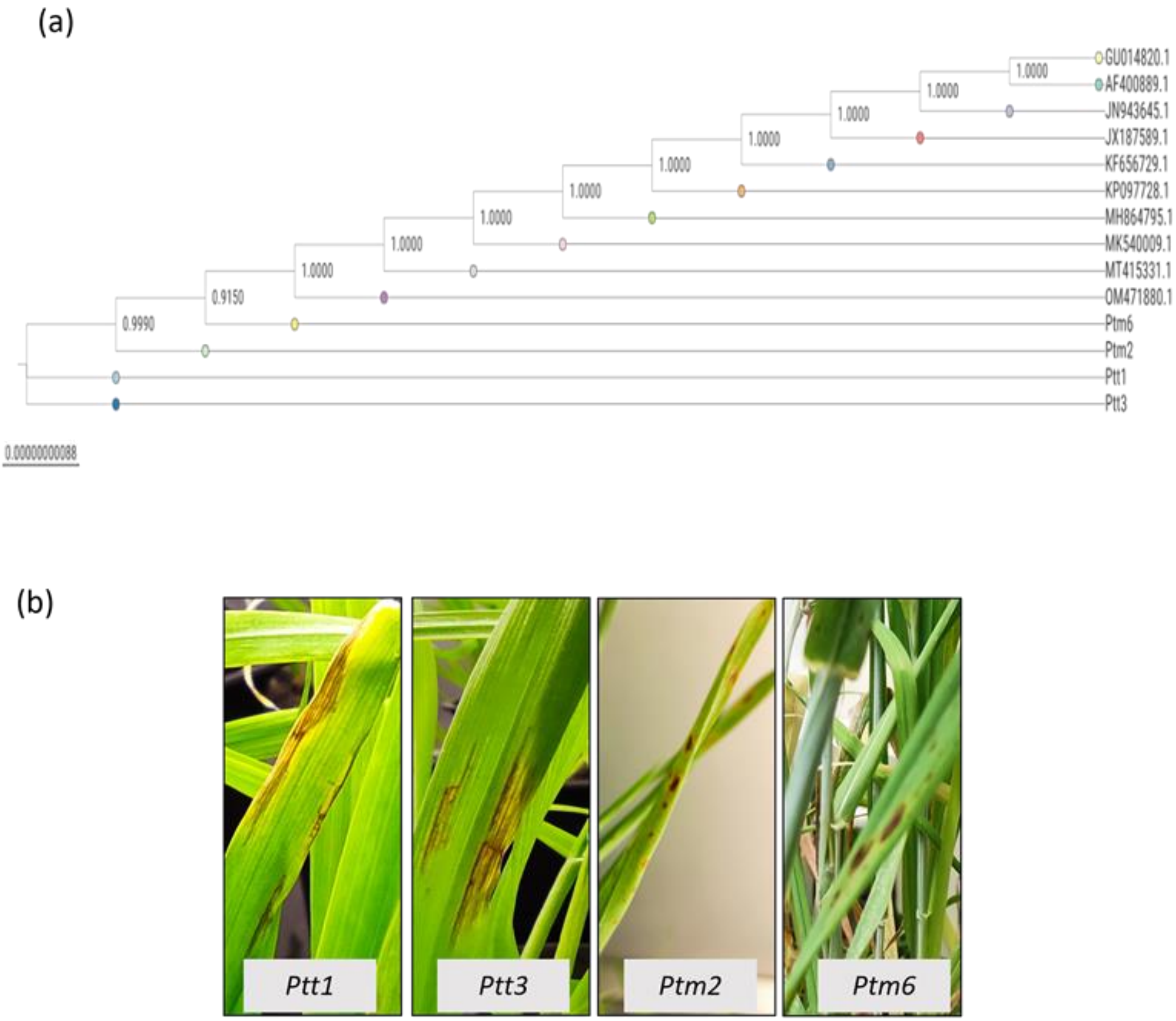

Supplementary figure S4: Integrated Phylogenetic and Symptom Analysis for *Pyrenophora teres* Identification: This figure combines (a) a phylogenetic tree, showing the genetic relationships of moroccan collected strains to reference ITS sequences, with (b) corresponding images of symptomatic barley leaves. These visual confirmations link the ITS barcoding results to the specific forms of *P. teres* responsible for the observed foliar symptoms.

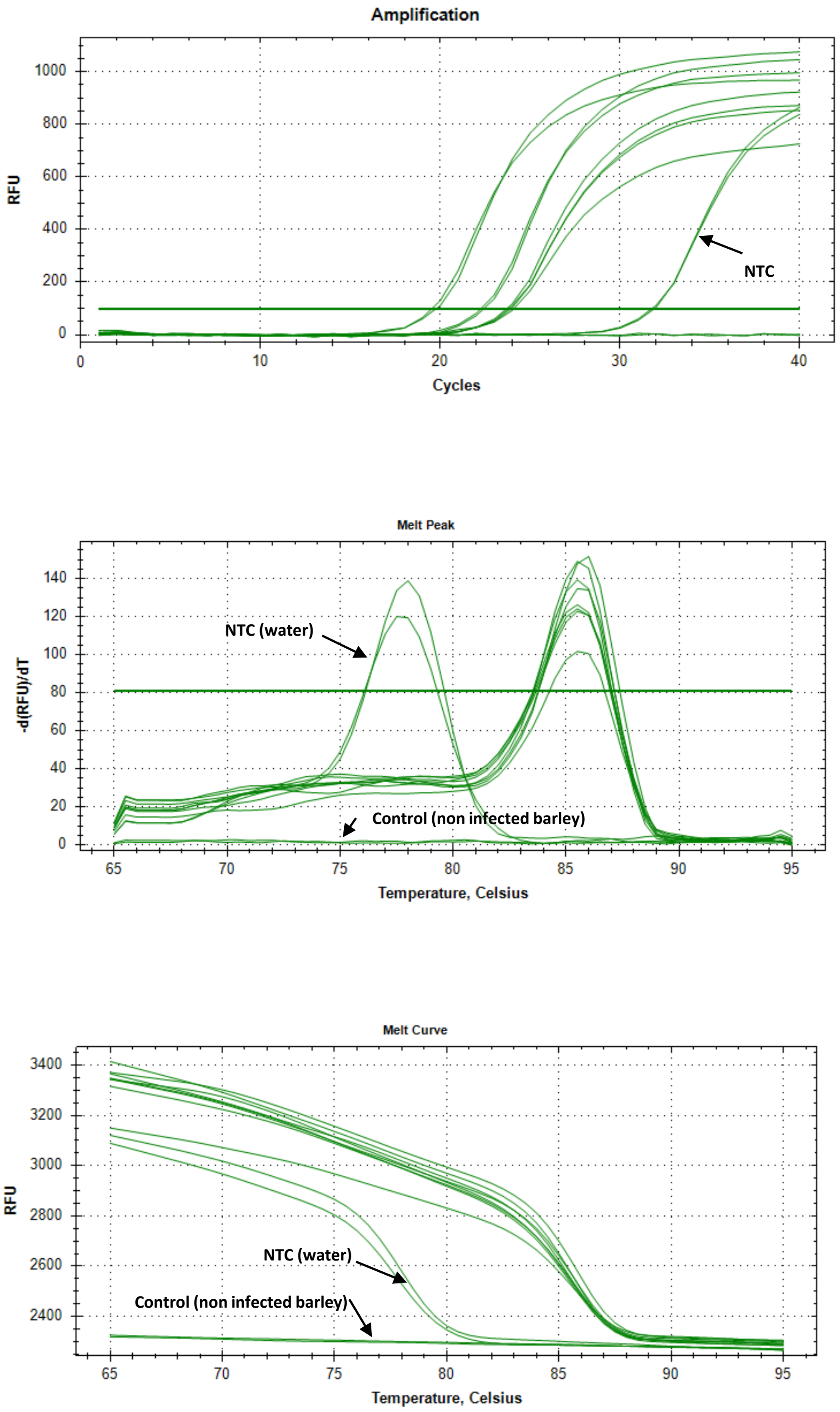

Supplementary figure S5: Amplification and melting curve of CONS1 primers. Primers were tested on DNA from 2 *Ptt* strains and 2 *Ptm* strains, from non-infected barley (control) and on no template control (NTC).

(a)

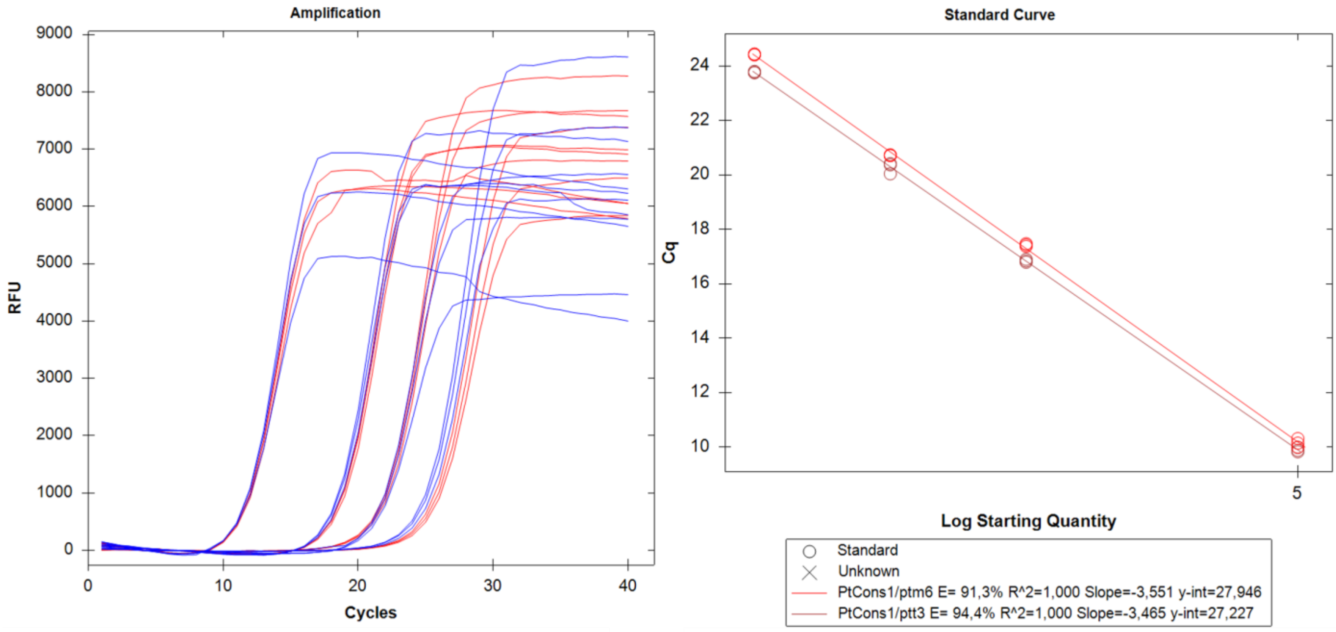

(b)

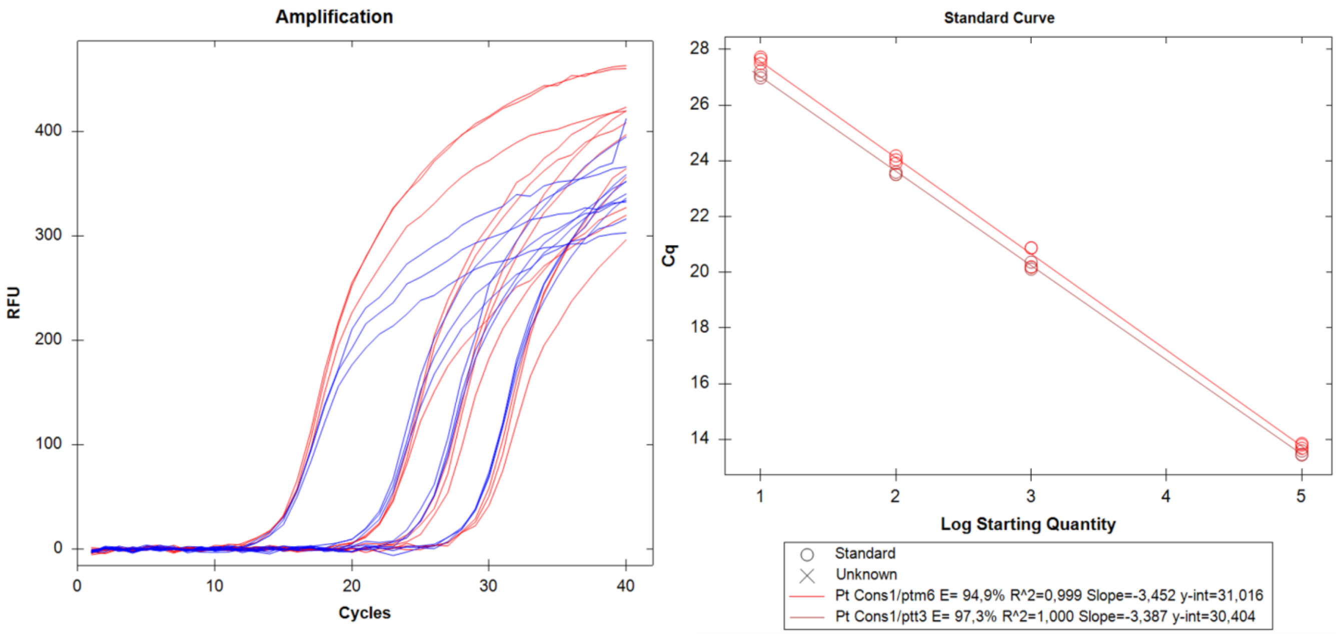

Supplementary figure S6: Primers efficiency in qPCR tested on *Ptt* and *Ptm* strains without (a) and with probe (b).

(a)

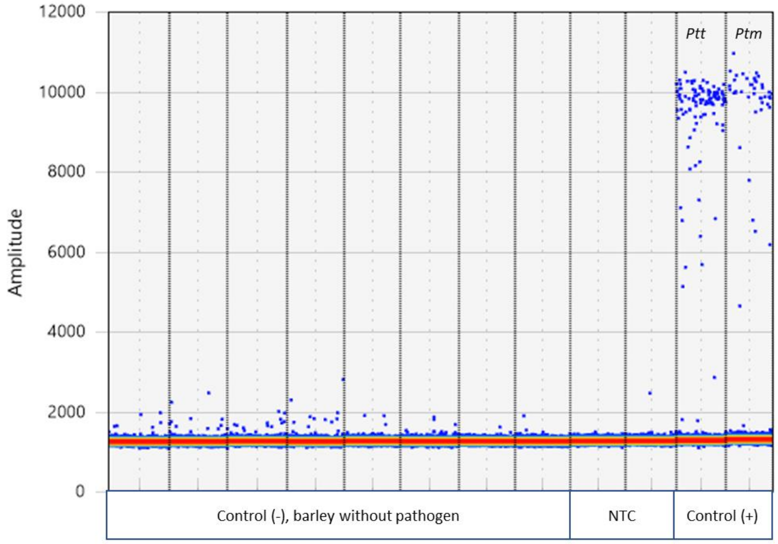

(b)

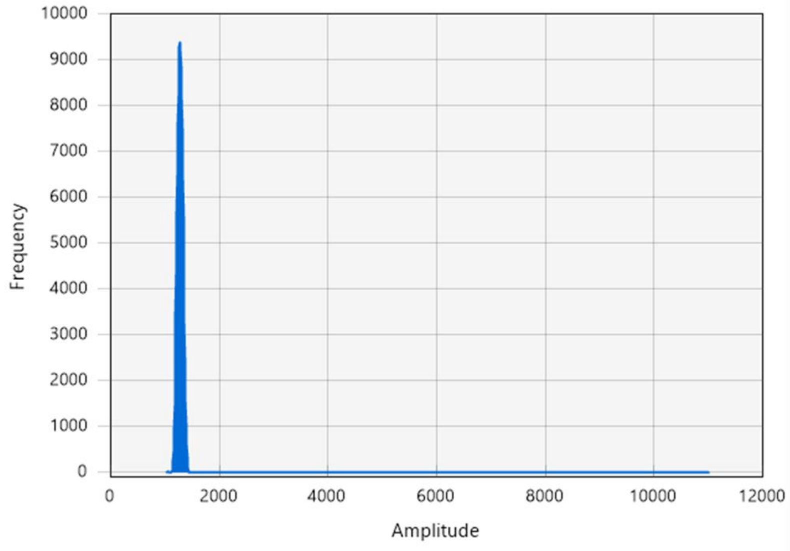

Supplementary figure S7: Specificity analysis of ddPCR. (a) 1D droplet spots of fluorescence amplitude for negative control (barley leaves without pathogen), NTC Non Treated Control (water) and positive controls (*Ptt* and *Ptm* strains). (b) Histogram result.

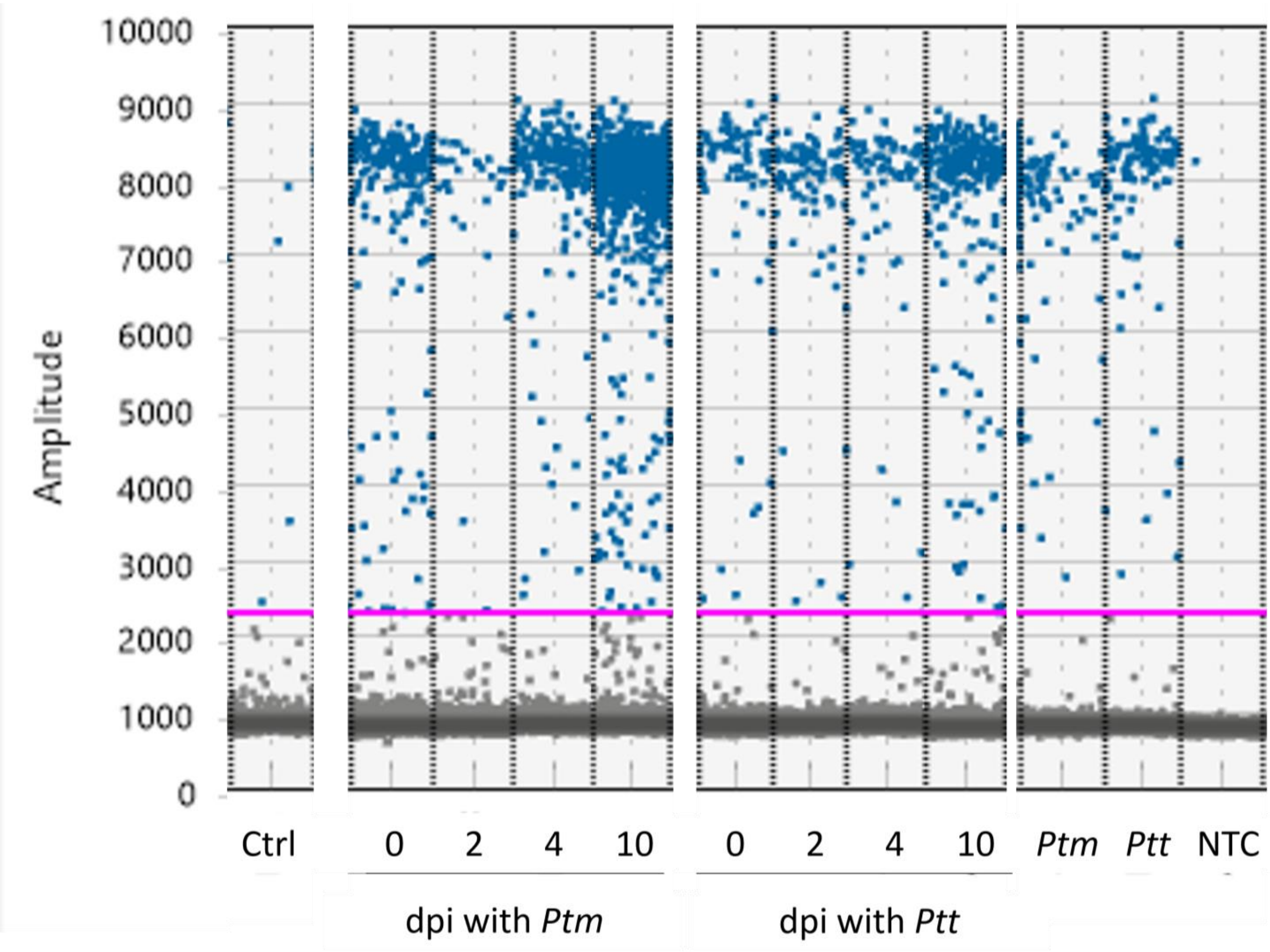

Supplementary figure S8: *In plan*ta detection of *Ptt* and *Ptm* by ddPCR. Ctrl = non infected plants (barley leaves without pathogen), NTC Non Treated Control (water) and positive controls (*Ptt* and *Ptm* strains).

Supplementary table S2: : Field sampling of fungi on *Hordeum vulgare* with molecular confirmation using Infection Response (IR) ratings based on Tekauz** (1985) and Neupane* (2015) scales at research farms across Morocco, 2019

Field code	Lab code	Host	Stage	LOCACTION	REGION	Sampling year	Infection Response	**Accession Numbers**	**Reference**	Similarity (ITS results)
Y6DH2	Ptm6	*Hordeum vulgare*	Adult	INRA Research farm-Jamaa Sahim	ABDA	2019	4.5*	-	GU014820.1	99,42%
Y19-42	Ptm2	*Hordeum vulgare*	Adult	3Km after MACHRAA BEN ABOU	MARRAKECH	2019	4*	-	JX187589.1	99,27%
SM 29-2	Ptt1	*Hordeum vulgare*	Adult	ICARDA Research farm-Marchouch	Rabat	2019	7**	-	KF656729.1	99,58%
Mor CC-B1	Ptt3	*Hordeum vulgare*	Adult	INRA Reasearch farm-Sidi Allal Tazi	Kenitra	2019	8**	-	MK540009.1	99,79%

Supplementary table S3: 22 *P.teres* available genomes (23/08/2023)

Organism Scientific Name	Organism Qualifier	Form	Taxonomy id	Assembly Name
Pyrenophora teres f. teres 0-1	strain: 0-1	Ptt	861557	PyrTer_1.0
Pyrenophora teres f. teres	isolate: 6A	Ptt	97479	ASM808672v1
Pyrenophora teres f. teres	isolate: 15A	Ptt	97479	ASM808675v1
Pyrenophora teres f. teres	isolate: BB25	Ptt	97479	ASM808678v1
Pyrenophora teres f. teres	isolate: FGOH04Ptt-21	Ptt	97479	ASM808684v1
Pyrenophora teres f. teres	strain: HRS9139	Ptt	97479	ASM972863v1
Pyrenophora teres f. teres	strain: HRS9122	Ptt	97479	ASM972864v1
Pyrenophora teres f. teres	strain: NB73	Ptt	97479	ASM972865v1
Pyrenophora teres f. teres	strain: NB29	Ptt	97479	ASM972866v1
Pyrenophora teres f. teres	strain: NB85	Ptt	97479	ASM972867v1
Pyrenophora teres f. maculata	isolate: DEN2.6	Ptm	97480	ASM1433475v1
Pyrenophora teres f. maculata	isolate: NZKF2	Ptm	97480	ASM1433477v1
Pyrenophora teres f. maculata	isolate: FGOB10Ptm-1	Ptm	97480	ASM1433479v1
Pyrenophora teres f. maculata	isolate: P-A14	Ptm	97480	ASM1433481v1
Pyrenophora teres	strain: LQA	PT	53485	ASM2002709v1
Pyrenophora teres Hybrid	ecotype: Australia, strain: WAC10721	Pth	53485	100x
Pyrenophora teres	strain: SNB172	PT	53485	ASM2550458v1
Pyrenophora teres	strain: Ptt12013	PT	53485	ASM2550460v1
Pyrenophora teres	strain: HRS10128	PT	53485	ASM2550461v1
Pyrenophora teres	strain: Ptm14015	PT	53485	ASM2550462v1
Pyrenophora teres f. maculata	isolate: SG1	PT	97480	ERZ478497
Pyrenophora teres f. teres	isolate: W1-1	Ptt	97479	PTTW11

Supplementary table S4: Primer and Probe Specifications for *Pyrenophora teres* qPCR and ddPCR Assays

	Sequence (5'->3')	Template strand	Length	Start	Stop	Tm	GC%	Amplicon length
Forward primer	TTACTTGCTGGACCAGCTGC	Plus	20	35	54	60,61	55	196
Reverse primer	CAGTTTGTCGTCCTTCTCGC	Minus	20	230	211	59,22	55	
Probe	(FAM)'C GCGGTTGGCGTTGACCGTGCC A(M C8E O)-	Plus	24	593	616	65,13	66,67	
